# Trends in Texas high school student enrollment in mathematics, science, and CTE-STEM courses

**DOI:** 10.1186/s40594-017-0063-6

**Published:** 2017-05-22

**Authors:** So Yoon Yoon, Johannes Strobel

**Affiliations:** 1 Institute for Engineering Education and Innovation (IEEI), College of Engineering, Texas A&M University, College Station, TX USA; 2 School of Information Science & Learning Technology (iSchool) College of Education, University of Missouri, Columbia, MO USA

**Keywords:** High school students, Mathematics, Science, Career and technical education (CTE), STEM, Course enrollment

## Abstract

**Background:**

In the context of Texas of the USA, House Bill 5 signifies a major policy shift requiring entering high school students starting in fall 2014 to choose an endorsement, like science, technology, engineering, and mathematics (STEM) being one of them, to provide students with earlier exposure to a coherent course sequence. As we barely understand students’ choices before the endorsement requirement, this study explored 6 years of data (2008–2013) on high school student enrollment rates in mathematics, science, and career and technical education (CTE)-STEM courses to set out the baseline of the trends in STEM course enrollment in Texas.

**Results:**

The enrollment rates of the STEM-related courses had wide variations by types of courses, gender, and race/ethnicity. Overall, student enrollment rates increased across time in selective and advanced mathematics, science, and CTE-STEM courses, which indicates a promising prospect for the STEM pipeline. However, there were exceptions in several courses with gender and racial/ethnic differences in the trends. Gender disparity was greater in advanced science courses than advanced mathematics courses, and collectively, gender gap in CTE-STEM courses increased greater than advanced mathematics and advanced science courses across years. While racial/ethnic differences were constant across years in both advanced mathematics and advanced science courses, the differences were rising in CTE-STEM courses in recent years.

**Conclusions:**

As little is known about students’ preferences in course-taking in STEM courses at the state level, the findings on the trends in students’ STEM course-taking, disaggregated by gender and race/ethnicity, can provide needed insights on what institutional K-12 changes would be effective for impacting the STEM pipeline.

While the demand for motivated students to enter science, technology, engineering, and mathematics (STEM) fields is at its highest, high school students’ interest in and readiness for pursuing these careers have been sluggish (e.g., ACT, 2006). The largest impact on STEM entrance is reported to be the intent to major in STEM, which is directly affected by high school students’ exposure to and achievement of mathematics and science courses (Wang, 2013). In the context of Texas of the USA, House Bill 5 signifies a major policy shift requiring entering high school students to choose an endorsement among five categories in fall 2014: (a) STEM, (b) Business and Industry, (c) Public Services, (d) Arts and Humanities, and (e) Multidisciplinary. Career and technical education (CTE) is one of the pathways to achieve the STEM endorsement, next to mathematics, science, and computer science. The goal for House Bill 5 is to provide students with earlier exposure and a coherent course sequence to increase preparedness and sustain interest in STEM careers (Mellor, Stoker, & Reese, 2015).

Given the increase in messaging on the value of STEM, we do not know how well the message is acted upon by high school students. As we barely understand students’ choices before the endorsement requirement, we need to set a baseline. Therefore, this study attempts to set out the baseline through analyses of trends in several years of mathematics, science, and CTE-STEM course enrollments (from 2008 to 2013 school years) in Texas (TX) prior to House Bill 5.

Among the several STEM-related coursework in high school, we particularly chose to focus on the CTE-STEM pathway out of two reasons. First, the CTE-STEM pathway includes the most explicit inclusion of engineering and technology courses, whereas the science, mathematics, and computer science pathways do not specifically require engineering courses for their completion. Second, CTE courses are designed to address students, who are more interested in entering the workforce after their high school degree. Therefore, CTE provides a lower entry point into STEM careers than a college-bound track.

## Background

### Roles of rigorous coursework

A strong relationship between rigorous coursework (taking advanced or challenging courses) in high school and postsecondary success is well established in the literature (ACT, 2005a, 2005b, 2006; Adelman, 1999, 2006; Yoon, Imbrie, & Reed, 2014). The literature, mostly utilized data from the National Educational Longitudinal Survey (NELS) and the High School and Beyond (HSB), revealed that high school students with rigorous coursework tended to have higher achievement and graduation rates in high school (Adelman, 1999, 2006), higher scores on college entrance exams (Adelman, 1999), better performance and higher graduation rates in college (Adelman, 1999, 2006; Schneider, Swanson, & Riegle-Crumb, 1998), and better earnings at work (Altonji, Blom, & Meghir, 2012; Rose & Betts, 2004). Particularly, taking rigorous courses in high school was a strong predictor of bachelor’s degree completion at a 4-year institution than any other factors, including students’ social economic status (SES) and gender (Adelman, 1999).

A study by Long et al. (2012), utilizing the data of Florida public high school students who graduated in 2002–2003, further investigated the effects of rigorous high school courses on students’ performance in both secondary and postsecondary education by employing a propensity score matching (PSM) method, which is a more elaborative technique than the traditional regression approach. By controlling the effects of background differences, they found that students who took rigorous courses by fall of tenth grade tended to achieve significantly higher scores on the Florida Comprehensive Assessment Test (FCAT), higher high school graduation rates, enrollment and GPA in a 4-year institution, and finally higher graduation rates with a bachelor’s degree. Taking rigorous courses was effective regardless of differences by gender, race/ethnicity, poverty, and students’ academic ability.

Particularly, Finkelstein and Fong (2008) investigated minority students’ course-taking patterns and preparation for postsecondary education in California’s public university systems. They addressed the importance of college preparation starting from ninth grade and fair access to standard college preparatory curriculum by high schools across the state. This is because students, who did not complete one year of college preparatory courses in ninth grade, tended to have lower completion rates of California’s public university system’s requirements, and even students with high GPAs, but from low performing high schools, tended to have high incompletion rates of the requirements by the end of high school. In addition, the gap of the incompletion rates of the requirements between minorities, including Hispanic and Black students, and White and Asian students was apparent in mathematics and laboratory science as well as English.

Utilizing the Class of 1997 students that graduated from Florida public high schools, Tyson et al. (2007) found gender and racial/ethnic disparities in students’ mathematics and science course-taking and their STEM degree attainments in college within the state of Florida. They revealed that female students tended to take low-level mathematics and science courses, and even though some female students took high-level courses, those students were less likely to obtain STEM degrees compared to male students. While Hispanic and Black students were less likely to take high-level mathematics and science courses compared to White students, the minority students who took the high-level courses tended to seek STEM degrees as likely as White students.

Further, Tyson (2011) modeled the effects of high school course grades on engineering students’ degree attainment in Florida universities during the 2002–2003 school year and found that high school AP Calculus grades were the most effective factor on Physics and Calculus II course performance in college, and students, who took AP Physics B and/or C, showed better performance in Physics and Calculus III in college. Tyson (2011) concluded that high school GPA, calculus, and physics course scores were effective measures to predict the prerequisite course performance in engineering.

In sum, even though the literature revealed a lack of rigor in the coursework of female and minority students, rigorous high school coursework was a strong predictor of the success in postsecondary courses beyond students’ gender and social economic status. However, as most of the literature utilized outdated data and focused on exploring the effects of a few high school courses on high school and postsecondary performance (e.g., Robinson, 2003), there has been a lack of research on the recent trends in students’ STEM-related course participation, particularly considering recent efforts by state and nation to increase students’ STEM interests (President’s Council of Advisors on Science Technology, 2012). In addition, as more efforts have been made in increasing women and minorities in the STEM pipeline, it is necessary to explore any subgroup differences in the trends of taking rigorous courses in high school.

### Roles of career and technical education

In the past, vocational education was known for job-training courses for students, who planned to directly enter the workforce after high school (Association for Career and Technical Education (ACTE), 2007; Lynch, 2000). However, along with the revision of the term from “vocational education” to “career and technical education” (CTE) by Carl D. Perkins Career and Technical Education Improvement Act of 2006, states and the nation initiated reforms of CTE courses for high school students to provide an opportunity to build up students’ interests and competencies in careers, so that they can have a smooth transition from secondary to postsecondary education (American Institute for Research (AIR), 2013; ACTE, 2007). Particularly, when quality CTE education aligns with rigorous coursework, it is expected that students perform better in academics in the secondary education (Israel, Myers, Lamm, & Galindo-Gonzalez, 2012) and earn higher salaries at work (Griffith & Wade, 2001). Therefore, as a link between secondary and postsecondary education, the roles of CTE became more important in preparing students for college and future careers than the past (ACTE, 2009; AIR, 2013).

**Fig. 1:**
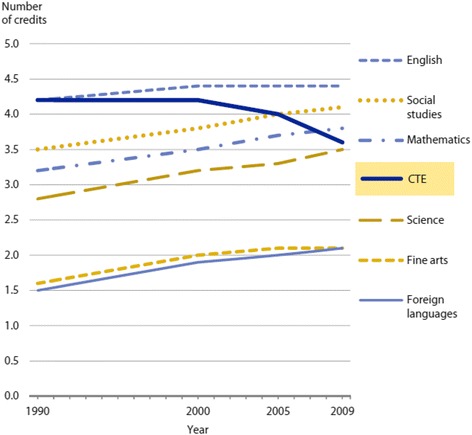
Average number of credits earned in each subject area by US public high school graduates in 1990, 2000, 2005, and 2009. Source: U.S. Department of Education (DoE) and National Center for Education Statistics (NCES) (2013, Fig. 1)

**Fig. 2:**
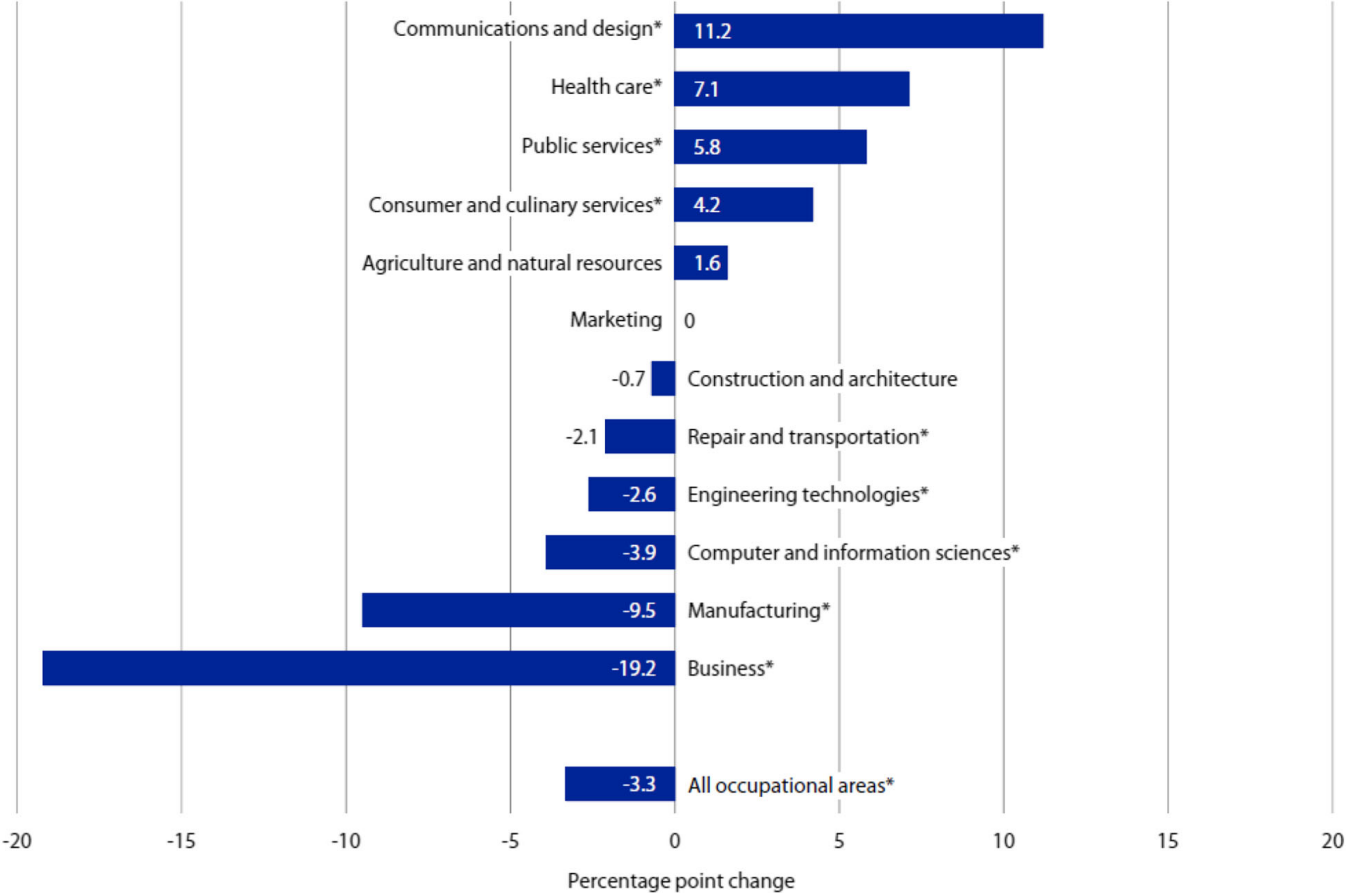
Change in the percentage of US public high school graduates’ CTE course credits by area between 1990 and 2009 (*p < .05). Source: U.S. DoE & NCES (2013, Fig. 2)

However, as shown in Fig. 1, the trends in CTE course-taking by U.S. public high school students were not promising, according to the data on average course credits from 1990 to 2009 by U.S. Department of Education (U.S. DoE) & National Center for Education Statistics (NCES) (U.S. DoE & NCES, 2013). While the average numbers of credits of major subjects earned per student have been steady or slightly increasing over time, the average number of CTE credits per student has been declining. Figure 2 shows the details of the change in the numbers of average credits by the CTE subjects. These changes are consistent in the findings by the report, utilizing the Educational Planning and Assessment System (EPASTM)—a longitudinal assessment system predicting students’ persistence and success in college—in that students’ interests in STEM majors were steadily declining across 10 years (ACT, 2006).

Under these circumstances in nation and by states, House Bill 5 is expected to bring substantial changes to Texas high school curricula and graduation requirements and significant effects in preparing students to be ready for college and career. Therefore, it is necessary to diagnose the student enrollment trends in the mathematics, science, and CTE-STEM courses to explore the effects of the changes and to provide evidence to prepare better support and policies to draw more students into STEM.

## Purpose of the study

This study aims to explore trends in Texas high school students’ preferences on STEM subjects by exploring students’ course enrollment data in Texas before House Bill 5, which was effective from the 2014-2015 school year. To do this, we utilized the 6 years of data (from 2008 to 2013 school years) from Texas Education Agency (TEA). Guiding research questions were as follows: (a) over a 6-year time frame, did Texas high school students take increasingly more STEM-related courses as measured by selective mathematics, science, and CTE-STEM courses, controlling for the effects of natural increase of population?; (b) how did the trends of students’ course enrollment differ by gender and race/ethnicity?; and (c) analyzing selective mathematics, science, and CTE-STEM courses, what trends in student enrollment can characterize students’ interest in STEM?; and (d) to what degree do students’ enrollment choices reflect college readiness trends in STEM?

## Method

### Setting

#### High school common curriculum in Texas

In Texas before the initiation of House Bill 5, there were three types of curriculum for high school graduation: (a) Minimum, (b) Recommended, and (c) Distinguished Advancement. Table 1 shows graduation requirements in mathematics and science courses for students entering ninth grade during 2007–2013 school years. For the Minimum track, students needed to take three mathematics courses, including Algebra I and Geometry, and two science courses including Biology and Integrated Physics and Chemistry (IPC). However, for the Recommended and Distinguished Advancement tracks, students needed to take four mathematics courses, including Algebra I, Geometry, and Algebra II, and four science courses, including Biology, Chemistry, and Physics.

**Table 1:** Mathematics and science courses by types of high school curriculum for graduation in Texas (for students entering ninth grade during 2007–2011 school years)

Program		Cr		Subject
Minimum	Mathematics	3	Required	Algebra I
			Required	Geometry
			Selective	Among 19 courses
	Science	2	Required	Biology
			Required	Integrated Physics and Chemistry (IPC)
			Substitute of IPC	Chemistry
			Substitute of IPC	Physics
Recommended and Distinguished Advancement	Mathematics	4	Required	Algebra I
			Required	Geometry
			Required	Algebra II^a^
			Selective	Among 18 courses
	Science	4	Required	Biology, AP Biology, or IB Biology
			Required	Chemistry, AP Chemistry, or IB Chemistry
			Required	Principles of Technology, Physics, AP Physics, or IB Physics
			Substitute	Integrated Physics and Chemistry (IPC)
			Selective	Among 25 courses

Source: Texas Education Agency (2014). Retrieved from http://www.tea.state.tx.us/graduation.aspx; http://ritter.tea.state.tx.us/rules/tac/chapter074/ch074f.html

*Cr* = credit

^a^Algebra II is not a requirement any more starting from Class of 2018 (2014 entrants) on the Recommended and Distinguished Advancement Tracks

#### CTE-STEM courses in Texas

Along with the common curriculum, Texas also offered career and technical education (CTE) programs in various areas. By selecting a program of their interests, students can take a sequence of courses with the CTE content that “is aligned with challenging academic standards and relevant technical knowledge and skills needed to prepare for further education and careers in current or emerging professions” (TEA, 2014, third paragraph). Among various CTE programs, the CTE-STEM program in Texas consists of 15 courses with varied credits, which ranged from 0.5 to 3.0 credits. Table 2 shows a list of CTE-STEM courses with course numbers titles, and credits, which was current as of the 2014–2015 school year.

**Table 2:** CTE-STEM courses offered in Texas as of the 2014–2015 school year

Course #	Course title	Credit
130.362	Concepts of Engineering and Technology	0.5–1
130.363	Biotechnology^R^	1–2
130.364	Advanced Biotechnology^R^	1
130.365	Engineering Design and Presentation^R^	1–2
130.366	Advanced Engineering Design and Presentation^P^	2–3
130.367	Engineering Mathematics^P^	1
130.368	Electronics^R^	1–2
130.369	Advanced Electronics^P^	2–3
130.370	Robotics and Automation^R^	1–2
130.371	Principles of Technology^P^	1 science
130.372	Scientific Research and Design^P^	1 science
130.373	Engineering Design and Problem Solving^P^	1 science
130.374	Practicum in Science, Technology, Engineering, and Mathematics	2–3
130.375	Principles of Engineering	1
130.376	Digital Electronics	1

Source: Texas Education Agency (2014). Retrieved from

http://ritter.tea.state.tx.us/rules/tac/chapter130/index.html

^R^ denotes that the course had recommended prerequisites; ^P^ denotes that the course had prerequisites

While five CTE-STEM courses had recommended prerequisites, five CTE-STEM advanced courses require prerequisites. Advanced Engineering Design and Presentation had a prerequisite of Engineering Design and Presentation; Engineering Mathematics had a prerequisite of Algebra II; Advanced Electronics had a prerequisite of Electronics; Principles of Technology had two prerequisites of one unit of high school science and Algebra I (which are general requirements of graduation for all high school students); Scientific Research and Design had a prerequisite of one unit of high school science; and Engineering Design and Problem Solving had four prerequisites of Geometry, Algebra II, Chemistry, and Physics.

According to the House Bill 5, from the class of 2018 high school students, there are several pathways for a student to earn a STEM endorsement, such as achieving four credits in mathematics, science, computer science, or CTE-STEM, respectively, on top of taking Algebra II, Chemistry, and Physics. For a CTE-STEM endorsement, a student needs to have a coherent course sequence of four or more credits, which consists of at least two courses in the same career cluster (CTE-STEM), including at least one advanced CTE course, which includes any course that is the third or higher course in a sequence.

### Population

The population of the study is grades 9–12 students in high schools in Texas from 2008 to 2013 school years. Based on the data from TEA, Table 3 shows demographic information of the grades 9 to 12 students in high school across 6 years. During the 6 years, the total number of the high school population has been increased about 8% from 1,292,587 to 1,407,868. Male students (~51%) were a slight majority. The student population is quite diverse in that the majority was Hispanic students (44~49%), which were close to half of the students’ population, followed by White (32~38%) and Black students (13~15%). While the number of Hispanic students has been increased, the numbers of Black and White students have been decreased across 6 years.

**Table 3:** Texas high school students’ demographic characteristics from 2008 to 2013 school years

Year	Female (%)	Male (%)	American Indian/Alaska Native (%)	Asian (%)	Black (%)	Hispanic (%)	Native Hawaiian/other Pacific (%)	White (%)	Multiracial (%)	Total *N*
2008–2009	48.9	51.1	0.4	3.7	14.7	43.5	−	37.8	−	1,292,587
2009–2010	48.8	51.2	0.5	3.4	13.8	45.5	0.1	35.2	1.4	1,318,403
2010–2011	48.9	51.1	0.5	3.6	13.5	46.5	0.1	34.3	1.5	1,335,617
2011–2012	48.8	51.2	0.5	3.7	13.3	47.4	0.1	33.4	1.6	1,358,435
2012–2013	48.8	51.2	0.4	3.7	13.1	48.3	0.1	32.6	1.7	1,381,979
2013–2014	48.8	51.2	0.4	3.8	13.0	48.9	0.1	32.0	1.7	1,407,868

For the 2008-2009 school year, there was no category for Native Hawaiian/other Pacific and Multiracial

### Data and analyses

The 6 years of data (from 2008 to 2013 school years) from TEA are open to the public and contain student enrollment records of all the courses offered in high school (grades 9–12) by gender and race/ethnicity. First, we identified all STEM-related courses for our analysis. Second, we set required mathematics and science courses for high school graduation as baseline courses since all students are required to achieve the credits. Third, we chose several selective mathematics and science courses in sequence recommended to be taken after the required courses. Fourth, among the selective courses, advanced courses at the college level were considered to be college preparatory courses in rigor. Fifth, CTE-STEM courses were identified across 6 years for the use of this study to explore the trends in student enrollment that can characterize students’ interests in STEM career.

In detail, in mathematics courses, Algebra I and Geometry were identified as required courses; Algebra II and Precalculus were selective; AP Calculus AB, AP Calculus BC, and AP Statistics were considered advanced mathematics courses as college preparatory courses. Among science courses, Biology was required; Chemistry, Physics, and Integrated Physics and Chemistry (IPC) were considered selective; and Advanced Biology courses including AP and IB, Advanced Chemistry courses including AP and IB, and Advanced Physics courses including AP Physics B, AP Physics C, and IB were considered advanced science courses as college preparatory courses. Among the current 15 CTE-STEM courses as of the 2014–15 school year in Table 2, the following six courses, except their advanced courses, consistently existed during the 2008 to 2013 school years with the same course title: Biotechnology, Electronics, Principles of Technology, Scientific Research and Design, Principles of Engineering, and Digital Electronics. However, when similar course titles existed across years, we also counted the enrollment rates of the courses, which matched with Advanced Biotechnology, Advanced Electronics, and Robotics and Automation.

Therefore, the course categories of our interests are a total of 29 course categories as listed in Table 4, including 7 in mathematics, 7 in science, and 15 in CTE-STEM. In addition, we collectively aggregated the data on all STEM-related courses offered in each year, including required, selective, and CTE-STEM courses to explore the overall trends as a whole as shown in the Figs. 1 and 2 in the report by U.S. DoE & NCES (2013).

**Table 4:** High school course category utilized in the study

Subject	Integrity	Course Category
Mathematics	Required	Algebra I, Geometry
	Selective	Algebra II, Precalculus
	Advanced	AP Calculus AB, AP Calculus BC, AP Statistics
Science	Required	Biology
	Selective	Chemistry, Physics, Integrated Physics and Chemistry (IPC)
	Advanced	Advanced Biology (including AP & IB), Advanced Chemistry (including AP & IB), Advanced Physics (including AP B, C & IB)
CTE-STEM	Selective	Concepts of Engineering and Technology, Biotechnology, Advanced Biotechnology, Engineering Design and Presentation, Advanced Engineering Design and Presentation, Engineering Mathematics, Electronics, Advanced Electronics, Robotics and Automation, Principles of Technology, Scientific Research and Design, Engineering Design and Problem Solving, Practicum in STEM, Principles of Engineering, Digital Electronics

*CTE* = career and technical education, *STEM* = science, technology, engineering, and mathematics

To explore the changes in the student enrollment in the STEM-related courses, a proportional ratio in percentage was calculated, using the enrolled number of students for a course per the total number of the student population, for each course category listed in Table 4 from 2008 to 2013 school years. In compliance with FERFA, student counts less than 5, not 0, were masked with −99 in the data from the TEA, so we considered the masked information counts of student between 1 and 4 with uncertainty. However, the differences caused by the uncertainty were smaller than 0.01% of the total student counts on course categories, so there were no significant changes in the results using proportional ratios in percentage caused by the uncertainty.

## Results

### Enrollment rates in required and selective mathematics courses by total and gender

**Fig. 3:**
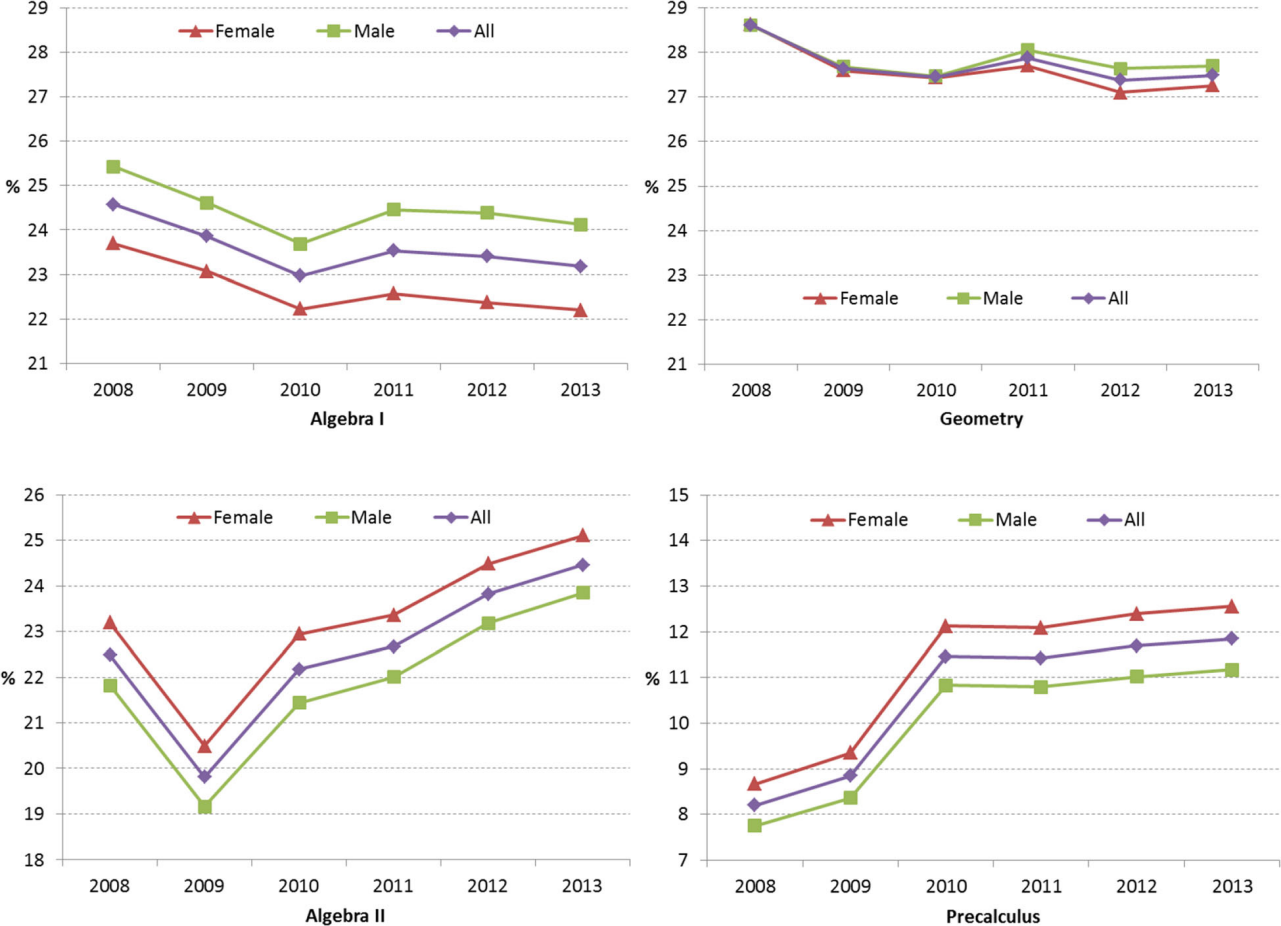
Required and selective mathematics course enrollment rates in percentage of all female, male, and total number of students from 2008 to 2013

Figure 3 shows enrollment rates in percentage of all female, male, and total number of students in two required and two selective mathematics courses. Overall, as presented in Table 5, student enrollment in Algebra I and Geometry in high school tended to decrease across 6 years (i.e., on average 0.23% per year and 0.19% per year, respectively), implying the increasing rates of students who complete the courses while in middle school or receive credits by exam (CBE). Further, we could observe slightly higher proportion of enrollment rates by male students in both courses, indicating that more female students tended to achieve the course credits prior to high school or through CBE while in high school. The trend was more apparent in Algebra I with a larger gender gap than Geometry as Table 5 shows the average differences between two genders across 6 years. However, the trends in both required courses turned to be opposite in two selective courses: Algebra II and Precalculus. Students enrolled in both courses tended to increase across 6 years, even though there was a drop in 2009 for Algebra II. However, the enrollment rates in Precalculus were about half of the ones in Algebra II as Algebra II is a required course for the Recommended and Distinguished Advancement tracks. Regarding gender differences, more female students than male students tended to enroll in the two selective courses and the trend was constant across the years with a similar gender gap.

**Table 5:** Six-year average enrollment rates and gain/loss of enrollment by gender in required and selective mathematics courses

	Algebra I		Geometry		Algebra II		Precalculus	
	Avg.	Gain/Loss	Avg.	Gain/Loss	Avg.	Gain/Loss	Avg.	Gain/Loss
All	23.59	−0.23	27.73	−0.19	22.57	0.33	10.58	0.61
Female	22.69	−0.25	27.61	−0.23	23.26	0.32	11.20	0.65
Male	24.45	−0.22	27.85	−0.15	21.91	0.34	9.99	0.57
Δ (Female–Male)	−1.76	−0.03	−0.24	−0.08	1.36	−0.02	1.21	0.08

Avg. = 6-year average enrollment rate in percentage; Gain = 6-year average gain (+) in enrollment rates in percentage; Loss = 6-year average loss (−) in enrollment rates in percentage

### Enrollment rates in required and selective science courses by total and gender

**Fig. 4:**
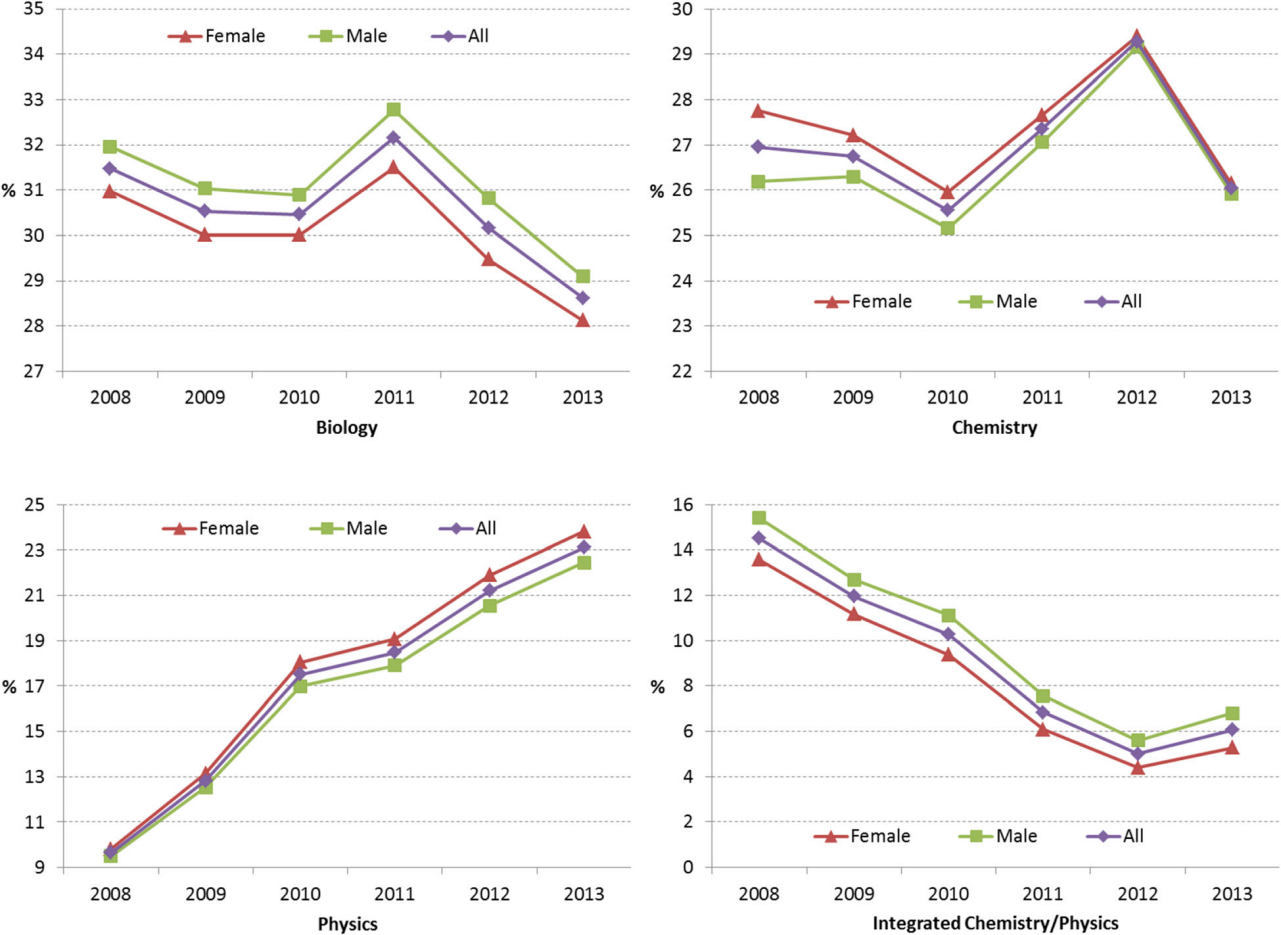
Required and selective science course enrollment rates in percentage of all female, male, and total number of students from 2008 to 2013

Figure 4 shows trends of student enrollment in three subject areas (biology, chemistry, and physics). As Biology was required, the enrollment rates (ranged from 28 to 33%) were higher than other subjects, followed by Chemistry (from 25 to 30%) and Physics (from 9 to 23%) (See Table 6 for the 6-year average enrollment rates and average gender differences in enrollment rates). While student enrollment rates in Physics tended to increase dramatically across 6 years, the trends of student enrollment in Biology and Chemistry across the years showed an interesting pattern. Particularly, the enrollment rates in Biology tended to decrease in the later 3 years (2011–2013). The enrollment rates in Integrated Physics and Chemistry (IPC) were continuously decreasing with a little bit turnover from 2012.

**Table 6:** Six-year average enrollment rates and gain/loss of enrollment by gender in required and selective science courses

	Biology		Chemistry		Physics		IPC	
	Avg.	Gain/Loss	Avg.	Gain/Loss	Avg.	Gain/Loss	Avg.	Gain/Loss
All	30.57	−0.48	26.99	−0.15	17.13	2.25	9.10	−1.41
Female	30.02	−0.48	27.36	−0.27	17.63	2.34	8.31	−1.38
Male	31.10	−0.48	26.63	−0.04	16.65	2.16	9.86	−1.43
Δ (Female–Male)	−1.08	0.00	0.72	−0.22	0.98	0.18	−1.55	0.05

*IPC =* Integrated Physics and Chemistry; Avg. = 6-year average enrollment rate in percentage; Gain = 6-year average gain (+) in enrollment rates in percentage; Loss = 6-year average decrease (−) in enrollment rates in percentage

Interestingly, more male students than female students enrolled in Biology, implying that more female students tended to achieve the course credits prior to high school or through CBE while in high school. In the early years, more female students than male students tended to enroll in Chemistry but the gap between two genders decreased. In Physics, there was no distinguishable gender gap in the enrollment in early years, but the gap became apparent in the subsequent years, with more female students than male students. More male students enrolled in Integrated Physics and Chemistry (IPC) than female students, and the gender gap was steady.

### Enrollment in six CTE-STEM courses by total and gender^1^

**Fig. 5:**
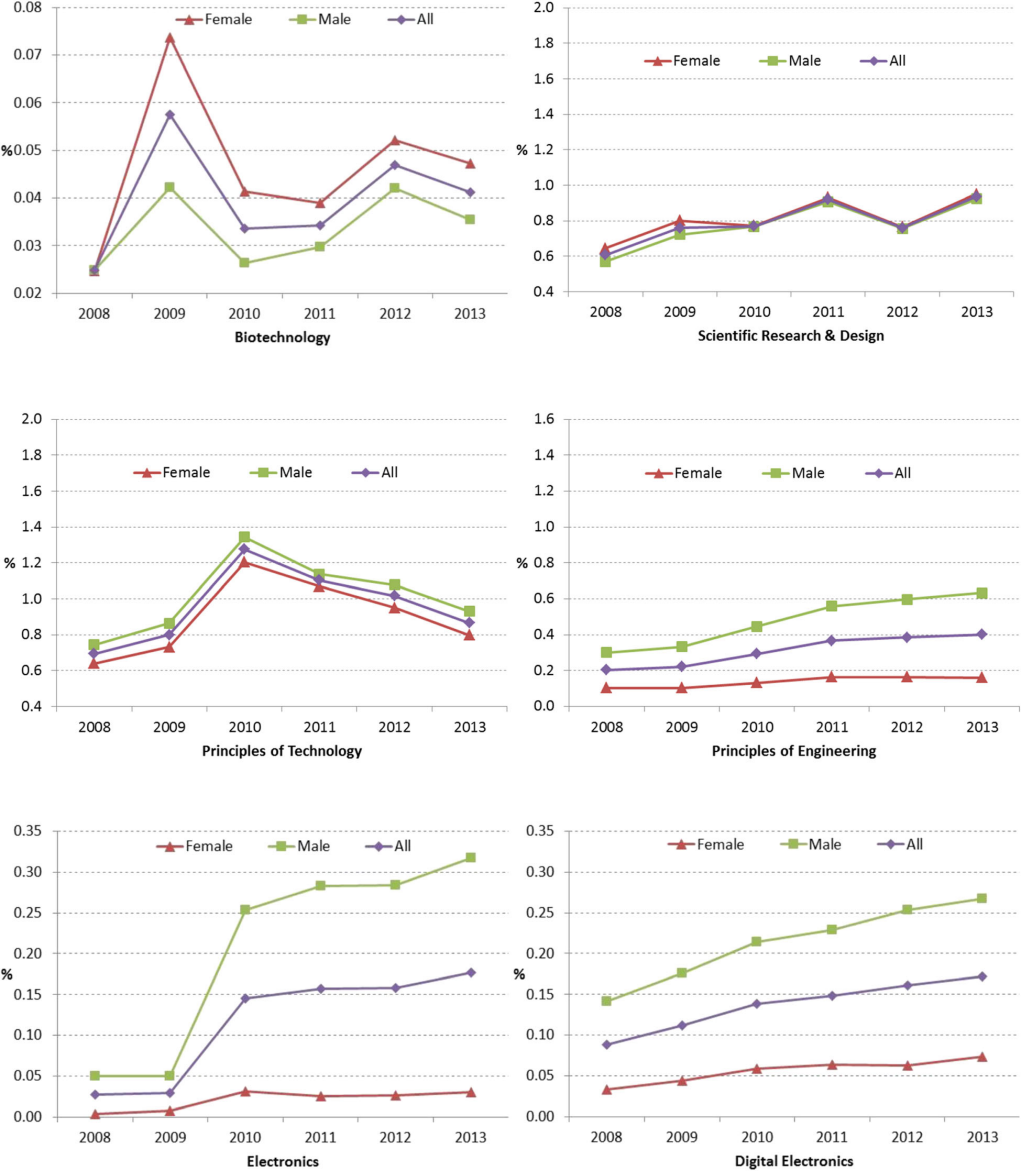
Six CTE-STEM course enrollment rates in percentage of all female, male, and total number of students from 2008 to 2013

Figure 5 shows the trend of enrollment in the six CTE-STEM courses (excluding advanced courses) continuously offered across 6 years. Among them, Principles of Technology had the highest enrollment rates (0.69~1.10% for all), followed by Scientific Research and Design (0.61~0.94% for all) and Principles of Engineering (0.20~0.40% for all) across years. Comparatively, the enrollment rates of Biotechnology, Electronics, and Digital Electronics were small, less than 0.5% of the total population.^2^ While the enrollment rates in Scientific Research and Design and Principles of Engineering were increasing across years (on average, 0.05% per year and 0.03% per year for all, respectively), the enrollment rates in Principles of Technology started to decrease from 2010.

Interestingly, in early years, more female students enrolled in Biotechnology and Scientific Research and Design than male students, but the gender gaps in the enrollment rates became smaller in Biotechnology and disappeared in later years in Scientific Research and Design, respectively. While more male students enrolled in Principles of Technology and Principles of Engineering than female students, the gender gaps were constant in Principles of Technology or even became larger in Principles of Engineering. Furthermore, the gender gap in Electronics and Digital Electronics became larger in the later years with more male students enrolled in those courses than female students.

### Overall enrollment rates in advanced mathematics, advanced science, and CTE-STEM courses by gender

**Fig. 6:**
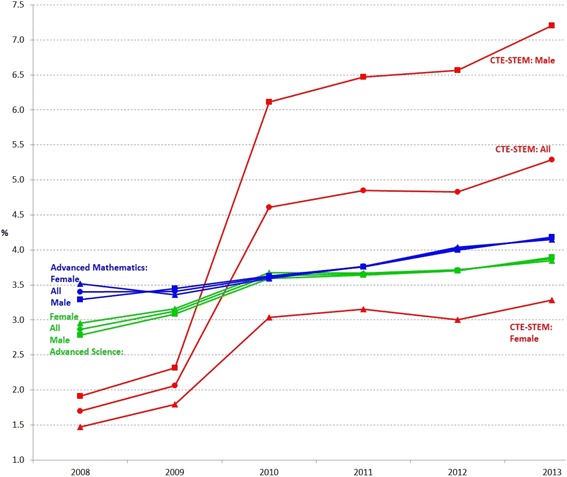
Advanced mathematics, advanced science, and CTE-STEM course enrollment rates in percentage of all female, male, and total number of students from 2008 to 2013

When the student enrollment data were aggregated by subject areas, such as advanced mathematics, advanced science, and CTE-STEM courses, Fig. 6 shows several notable trends. First, the overall enrollment rates across three subject areas have increased across years, which is a positive sign (see the 6-year average gains in enrollment rates in percentage for all three subject areas as shown in Table 7). Second, compared to advanced mathematics and science courses, the enrollment rates in CTE-STEM courses spiked in the later years and were continuously increasing (on average, 0.60% per year for all students), which implies increased student interests in CTE-STEM courses. Third, the collective gender gaps in advanced mathematics and science course have been decreasing (on average, −0.04% per year for both advanced mathematics and science courses), but the collective gender gap in CTE-STEM courses was increasing (on average, 0.60% per year) with more enrollments of male students.

**Table 7:** Six-year average enrollment rates and gain/loss of enrollment by gender in advanced mathematics, advanced science, and all CTE-STEM courses

	Advanced Mathematics		Advanced Science		All CTE-STEM	
	Avg.	Gain/Loss	Avg.	Gain/Loss	Avg.	Gain/Loss
All	3.73	0.13	3.48	0.17	3.89	0.60
Female	3.74	0.10	3.50	0.15	2.62	0.30
Male	3.72	0.15	3.45	0.18	5.10	0.88
Δ (Female–Male)	0.02	−0.04	0.05	−0.04	−2.47	−0.58

Avg. = 6-year average enrollment rate in percentage; Gain = 6-year average gain (+) in enrollment rates in percentage; Loss = 6-year average decrease (−) in enrollment rates in percentage

**Fig. 7:**
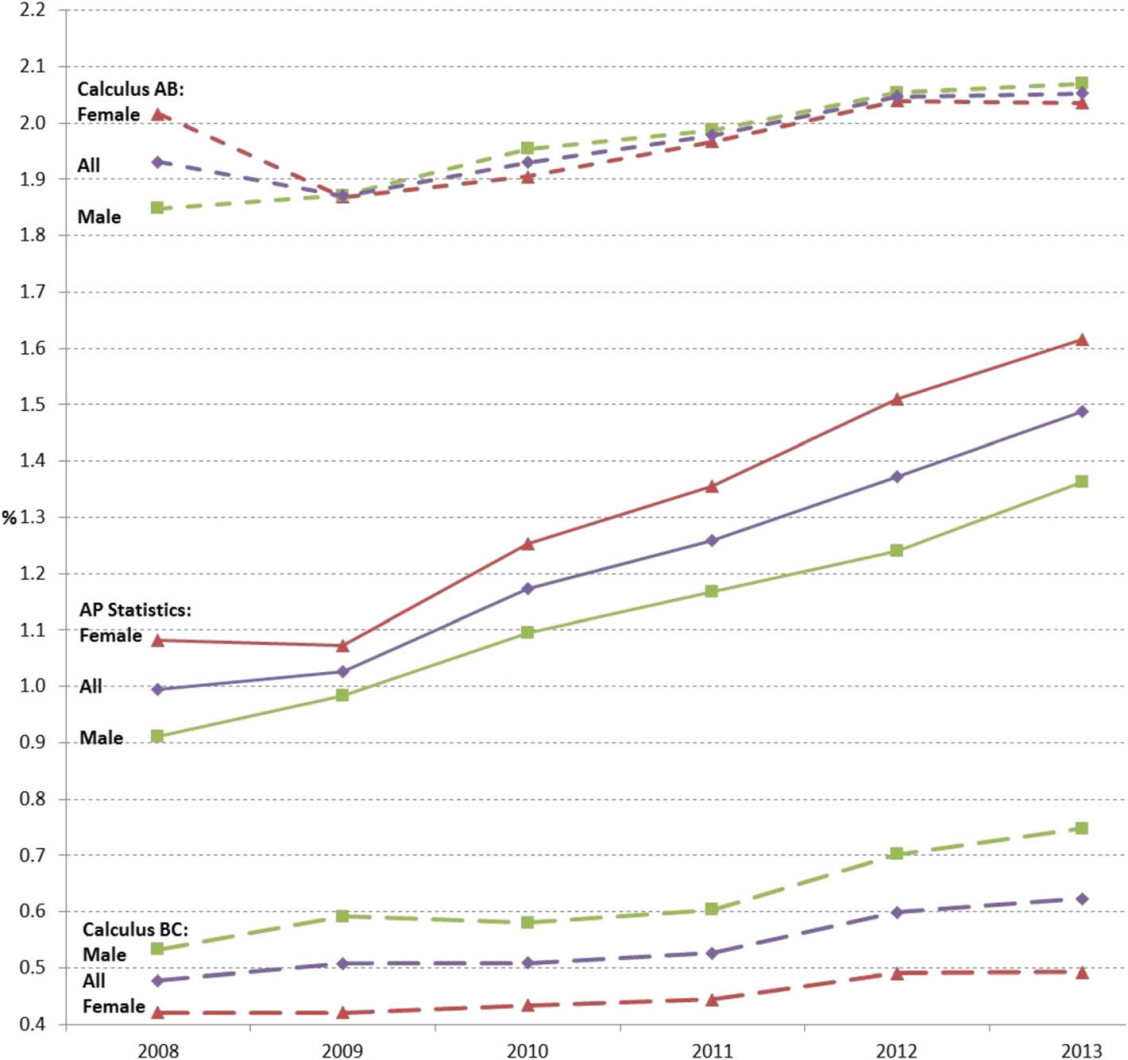
Advanced mathematics course enrollment rates in percentage of all female, male, and total number of students from 2008 to 2013

When the data were disaggregated by each course in mathematics as shown in Fig. 7, a small portion of students took the advanced mathematics courses, which were around 2.0% in AP Calculus AB, around 0.5% in AP Calculus BC, and around 1.2% in AP Statistics (See 6-year average enrollment rates in Table 8), compared to the required and selective mathematics courses as shown in Fig. 3. While enrollment rates were gradually increasing, more female students tended to enroll in AP Statistics (the 6-year average difference in the enrollment rate = 0.19%), and more male students tended to enroll in AP Calculus BC (0.18%).

**Table 8:** Six-year average enrollment rates and gain/loss of enrollment by gender in AP Calculus AB, AP Calculus BC, and AP Statistics

	AP Calculus AB		AP Calculus BC		AP Statistics	
	Avg.	Gain/Loss	Avg.	Gain/Loss	Avg.	Gain/Loss
All	1.97	0.02	0.54	0.02	1.22	0.08
Female	1.97	0.00	0.45	0.01	1.31	0.09
Male	1.96	0.04	0.63	0.04	1.13	0.08
Δ (Female–Male)	0.01	−0.03	−0.18	−0.02	0.19	0.01

Avg. = 6-year average enrollment rate in percentage; Gain = 6-year average gain (+) in enrollment rates in percentage; Loss = 6-year average decrease (−) in enrollment rates in percentage

**Fig. 8:**
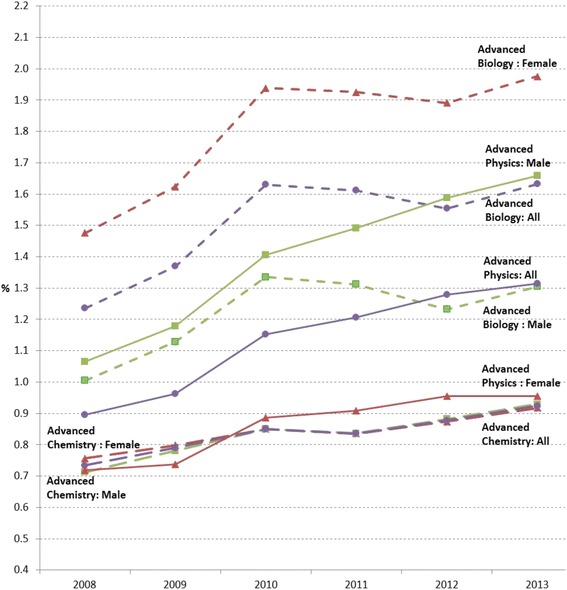
Advanced science course enrollment rates in percentage of all female, male, and total number of students from 2008 to 2013

Similarly, in advanced science courses, the enrollment rates were relatively small compared to the required and selective science courses, as shown in Fig. 8. As a whole, there were more students enrolled in Advanced Biology (6-year average of 1.51% for all students), followed by Advanced Physics (1.14%) and Advanced Chemistry (0.84%). As the enrollment rate in Physics was increasing across the years, the trend in Advance Physics was similar. In addition, more female students enrolled in Advanced Biology than male students, similar portions of male and female students enrolled in Advanced Chemistry, and more male students enrolled in Advanced Physics than female students (See Table 9 for the 6-year averages of enrollment rates by gender). The gender gaps in the advanced science courses seem to well reflect the existing gender gaps in postsecondary education by major (e.g., Riegle-Crumb & King, 2010; Riegle-Crumb & Moore, 2013, 2014).

**Table 9:** Six-year average enrollment rates and gain/loss of enrollment rates by gender in advanced biology, advanced chemistry, and advanced physics

	Advanced Biology		Advanced Chemistry		Advanced Physics	
	Avg.	Gain/Loss	Avg.	Gain/Loss	Avg.	Gain/Loss
All	1.51	0.07	0.84	0.03	1.14	0.07
Female	1.81	0.08	0.84	0.03	0.86	0.04
Male	1.22	0.05	0.83	0.04	1.40	0.10
Δ (Female–Male)	0.59	0.03	0.01	−0.01	−0.54	−0.06

Avg. = 6-year average enrollment rate in percentage; Gain = 6-year average gain (+) in enrollment rates in percentage; Loss = 6-year average decrease (−) in enrollment rates in percentage

**Fig. 9:**
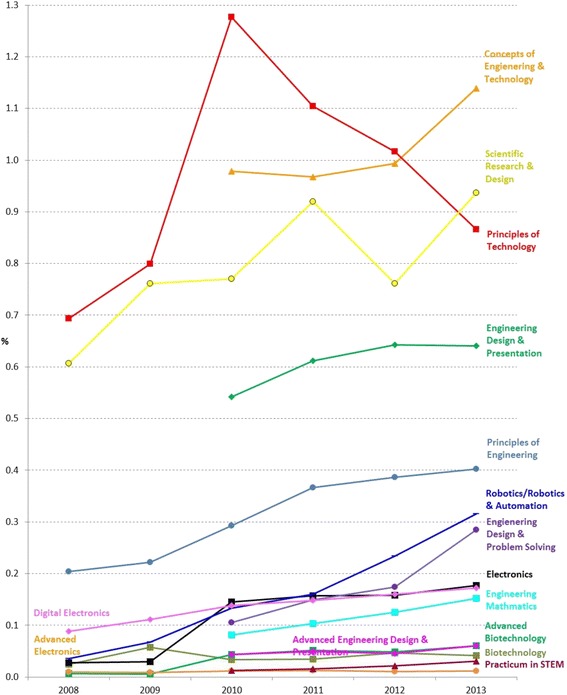
15 CTE-STEM course enrollment rates in percentage of total number of students from 2008 to 2013

Figure 9 shows the trends of student enrollment rates in percentage of total number of students in the 15 CTE-STEM courses across 6 years. Overall, the enrollment rates of most CTE-STEM courses were increasing except Principles of Technology. The top three popular CTE-STEM courses were Concepts of Engineering and Technology, Scientific Research and Design, and Principles of Technology, followed by Engineering Design and Presentation and Principles of Engineering.

On the one hand, as Concepts of Engineering and Technology is a recommended prerequisite of four CTE-STEM courses (i.e., Biotechnology, Engineering Design and Presentation, Electronics, and Robotics and Automation), the enrollment rates across time were relatively high compared to other courses and were increasing. On the other hand, while Scientific Research and Design had one prerequisite and Principles of Technology had two prerequisites, their enrollment rates across time were also relatively high. This is because their prerequisites are the courses required for all students to graduate from high school, so students are easily able to meet the requirements. Interestingly, even though Engineering Design and Problem Solving had four prerequisites, the enrollment rate in the last year was higher than two courses, Digital Electronics and Practicum in STEM, with no prerequisites and three courses, Electronics, Biotechnology, and Advanced Biotechnology, with recommended prerequisites.

### Enrollment rates in required and selective mathematics courses by race/ethnicity

**Fig. 10:**
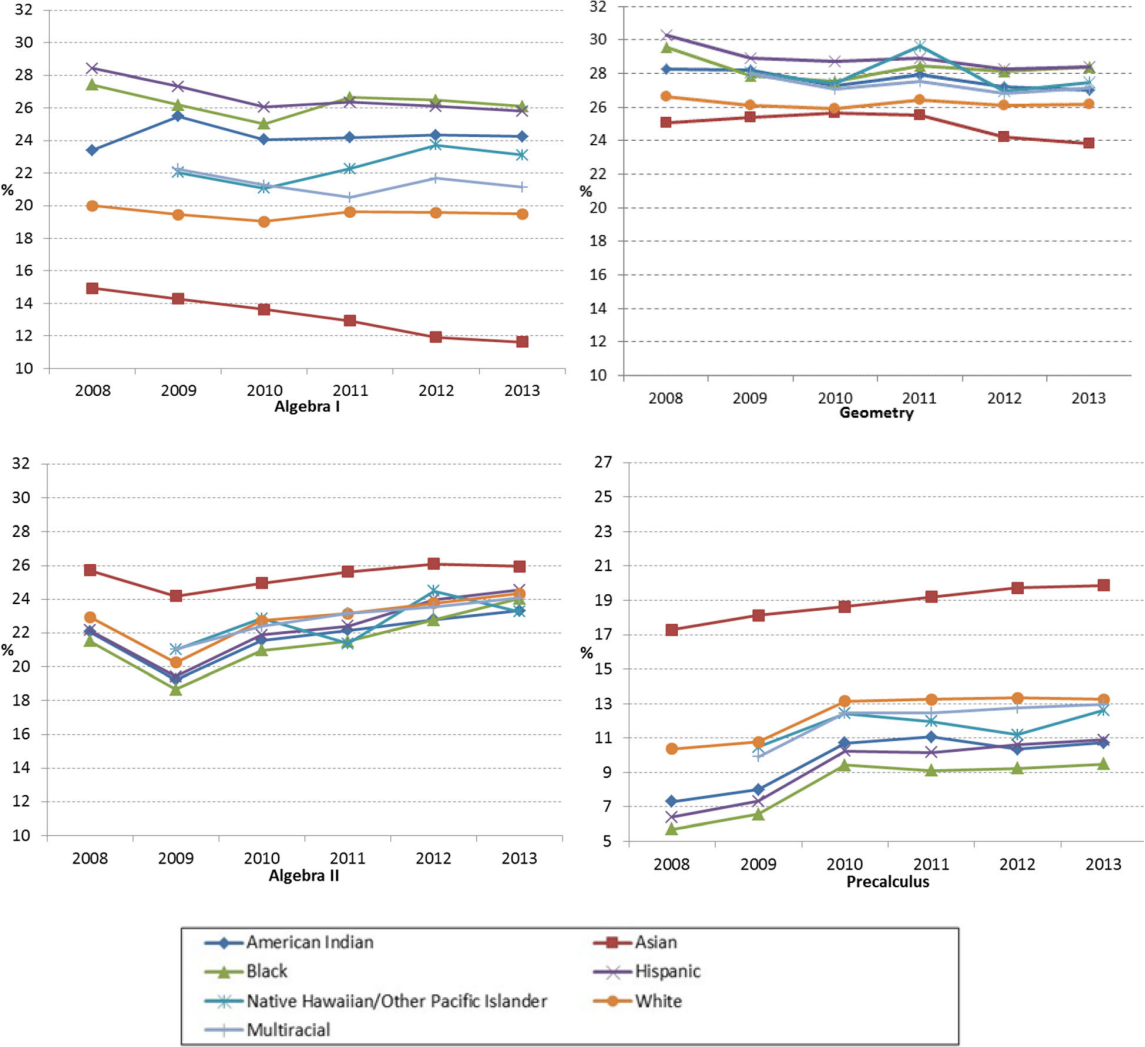
Required and selective mathematics course enrollment rates in percentage of total number of students in each racial/ethnic group from 2008 to 2013

Figure 10 delineates the trends in enrollment rates of the required and selective mathematics courses by race/ethnicity. As Table 10 shows the 6-year average enrolment rates from 2008 to 2013, compared to other race/ethnicity, the low enrollment rates of Asian students, followed by White students in Algebra I and Geometry, implies that they already achieved the credits while in middle school or through CBE. Overall, the enrollment rates of the selective courses, Algebra II and Precalculus, slightly increased in all race/ethnic groups (on average, 0.3% per year for Algebra II and 0.6% per year for Precalculus). Compared to other race/ethnic groups, Black students showed the lowest enrollment rates in Precalculus across 6 years (on average, 8.3%), followed by Hispanic students (on average, 9.3%).

**Table 10:** Six-year average enrollment rates and gain/loss of enrollment by race/ethnicity in required and selective mathematics courses

	Algebra I		Geometry		Algebra II		Precalculus	
	Avg.	Gain/Loss	Avg.	Gain/Loss	Avg.	Gain/Loss	Avg.	Gain/Loss
American Indian/Alaska Native	24.28	0.14	27.65	−0.20	21.86	0.21	9.69	0.57
Asian	13.22	−0.55	24.95	−0.21	25.43	0.04	18.81	0.43
Black	26.31	−0.22	28.32	−0.20	21.58	0.42	8.26	0.63
Hispanic	26.67	−0.44	28.92	−0.32	22.41	0.40	9.27	0.75
Native Hawaiian/other Pacific Islander	22.43	3.85	27.90	4.58	22.60	3.88	11.74	2.10
White	19.52	−0.09	26.22	−0.08	22.86	0.23	12.35	0.48
Multiracial	21.36	3.52	27.32	4.52	22.85	4.01	12.11	2.16
All	23.59	−0.23	27.73	−0.19	22.57	0.33	10.58	0.61

Avg. = 6-year average enrollment rate in percentage; Gain = 6-year average gain (+) in enrollment rates in percentage; Loss = 6-year average decrease (−) in enrollment rates in percentage

### Enrollment rates in required and selective science courses by race/ethnicity

**Fig. 11:**
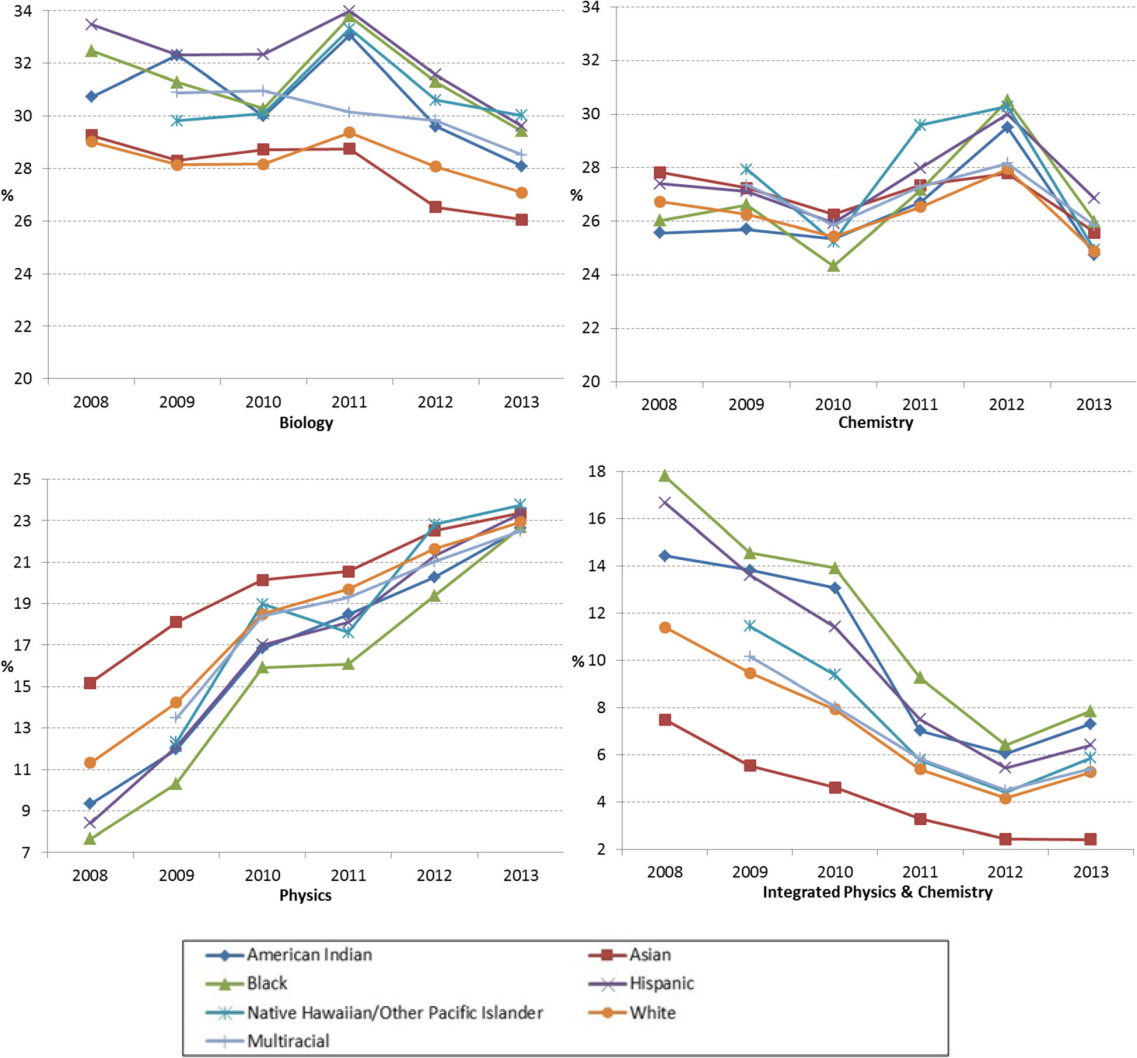
Required and selective science course enrollment rates in percentage of total number of students in each racial/ethnic group from 2008 to 2013

Like the trends in the required courses (Algebra I and Geometry) in mathematics, Fig. 11 shows that Asian and White students showed relatively low enrollment rates in Biology (on average 27.9% for Asian and 28.3% for White) compared to other racial/ethnic groups, implying credits achieved during middle school or through CBE. As shown in Table 11, the decrease in the enrollment rates in Biology (on average −0.5% per year for all) indicates that more students achieved the credits prior to high school. The enrollment rates by race/ethnicity were comparable in Chemistry, but there was a decrease in 2013. In Physics, enrollment rates were increasing across years (on average 2.3% for all) and the gaps among racial/ethnic groups were also narrowing. In Integrated Physics and Chemistry (IPC), the enrollment rates were decreasing across 6 years except 2013 for all racial/ethnic groups (on average, −1.41% per year). The relatively low enrollment rates by Asian and White students in IPC indicate that they tended to take other science courses or advanced science courses instead of IPC.

**Table 11:** Six-year average enrollment rates and gain/loss of enrollment by race/ethnicity in required and selective science courses

	Biology		Chemistry		Physics		IPC	
	Avg.	Gain/Loss	Avg.	Gain/Loss	Avg.	Gain/Loss	Avg.	Gain/Loss
American Indian/Alaska Native	30.63	−0.44	26.27	−0.14	16.59	2.21	10.28	−1.19
Asian	27.94	−0.54	27.00	−0.37	19.98	1.36	4.29	−0.85
Black	31.42	−0.51	26.78	−0.01	15.34	2.51	11.63	−1.66
Hispanic	32.22	−0.64	27.55	−0.09	16.71	2.49	10.17	−1.71
Native Hawaiian/other Pacific Islander	30.77	5.00	27.60	4.15	19.09	3.96	7.37	0.98
White	28.31	−0.32	26.30	−0.31	18.06	1.94	7.26	−1.02
Multiracial	30.07	4.75	26.91	4.31	18.94	3.75	6.78	0.90
All	30.57	−0.48	26.99	−0.15	17.13	2.25	9.10	−1.41

*IPC=* Integrated Physics and Chemistry; Avg. = 6-year average enrollment rate in percentage; Gain = 6-year average gain (+) in enrollment rates in percentage; Loss = 6-year average loss (−) in enrollment rates in percentage

### Collective enrollment rates in advanced mathematics, advanced science, and CTE-STEM courses by race/ethnicity

**Fig. 12:**
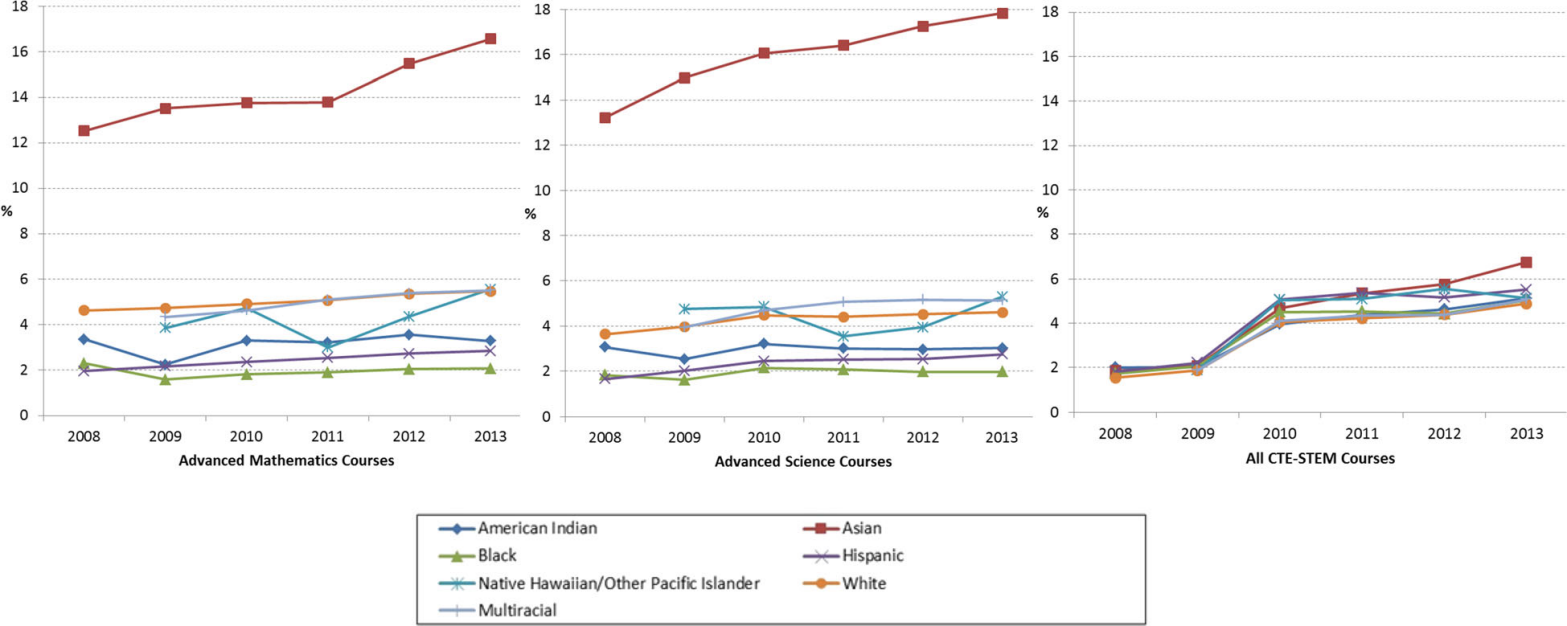
Advanced mathematics, advanced science, and CTE-STEM course enrollment rates in percentage of total number of students in each racial/ethnic group from 2008 to 2013

Figure 12 shows student enrollment rates aggregated by subject areas, such as advanced mathematics, advanced science, and CTE-STEM courses, but disaggregated by race/ethnicity. As we could expect, Asian students showed the highest enrollment rates across years in advanced mathematics and advanced science courses (on average, 14.3 and 16.0%, respectively), and Black students showed the lowest enrollment rates (on average, 2.0%) followed by Hispanic students (on average, 2.4%) (See Table 12). Interestingly, in CTE-STEM courses, there were no apparent racial/ethnic group gaps in early years, but the gaps became apparent in later years, with soaring in Asian students’ enrollment in 2013 (6.8%).

**Table 12:** Six-year average enrollment rates and gain/loss of enrollment by race/ethnicity in advanced mathematics, advanced science, and CTE-STEM courses

	Advanced Mathematics		Advanced Science		All CTE-STEM	
	Avg.	Gain/Loss	Avg.	Gain/Loss	Avg.	Gain/Loss
American Indian/Alaska Native	3.16	−0.01	2.97	−0.01	3.69	0.52
Asian	14.28	0.67	15.96	0.77	4.41	0.82
Black	1.95	−0.04	1.93	0.02	3.72	0.55
Hispanic	2.43	0.15	2.32	0.18	4.19	0.62
Native Hawaiian/other Pacific Islander	4.30	0.93	4.48	0.88	4.54	0.85
White	5.03	0.14	4.28	0.16	3.49	0.56
Multiracial	5.00	0.92	4.80	0.86	3.95	0.85
All	3.73	0.13	3.48	0.17	3.89	0.60

Avg. = 6-year average enrollment rate in percentage; Gain = 6-year average gain (+) in enrollment rates in percentage; Loss = 6-year average loss (−) in enrollment rates in percentage

## Discussion

Using the student enrollment data on STEM-related courses over a 6-year period, the findings of this study provide a basis for exploring high school students’ interests in STEM, prior to House Bill 5 that signifies a major policy shift in Texas high school students’ course-taking. In sum, the results showed wide variations in the enrollment rates on the STEM-related courses by types of courses, gender, and race/ethnicity. While most courses showed increased enrollment rates, which indicate a promising prospect for the STEM career pathways, there were exceptions in several courses and gender and racial/ethnic differences in the trends. Based on the findings, we identified several notable characteristics in the trends of student course-taking in mathematics, science, and CTE-STEM courses, so addressed each characteristics one by one with discussion.

### STEM course preparation for college seemed to start from middle school

While Finkelstein and Fong (2008)—using California student data—revealed the importance of course preparation from ninth grade for college, the Texas high school student data showed that students’ course preparation already started in middle school by achieving the required course credits in high school, such as Algebra I, Geometry, Biology, and Chemistry. In addition, the trends for early credit achievement have become stronger in recent years as indicated by the 6-year average loss rates of enrollment in Tables 5 and 6. Particularly, the trend was apparent in mathematics and more significant for female students, Asian, and/or White students.

Considering the mathematics course options available in high school, which include seven courses (Algebra I, Geometry, Algebra II, Precalculus, AP Calculus AB, AP Calculus BC, and AP Statistics), it is hard for students to take them all in the 4-year high school curriculum, implying the need of students’ advancement in mathematics in middle school to meet the prerequisite requirements for high-level courses (Catsambis, 1994). As middle school is known to be an ideal time for students to plan about their college and career (Wimberly & Noeth, 2006), if students are interested in STEM, appropriate guidelines are necessary to help them identify course options during middle school through high school. In addition, as ACT (2006) suggested for policy makers and educational leaders, it would be good to include a longitudinal student progress assessment in mathematics to identify and improve students’ readiness for college and provide a support and/or awareness program to reduce gender and ethnic/racial gaps in the course preparedness from middle school.

### Overall, student enrollment rates increased across years in selective and advanced mathematics, advanced science, and CTE-STEM courses

As shown in Figs. 3, 4, 5, 6, and 7, over a 6-year time frame, overall Texas high school student enrollment rates were increasing in selective mathematics courses (e.g., Algebra II and Precalculus), selective science course (e.g., Physics) except Integrative Physics & Chemistry (IPC), advanced mathematics courses, advanced science courses, and CTE-STEM courses when the effects of natural increase of population were controlled in enrollment rates. Even though the proportions of students taking the advanced mathematics, advanced science, and CTE-STEM courses (less than 5%) are relatively smaller than ones of selective mathematics and science courses, the trends are promising as it reflects a continuous increase of students’ interests in STEM across years. However, it was apparent that most students (more than 95%) did not take advanced mathematics and advanced science courses as shown in Fig. 6. Therefore, reinforced educational strategies and policies are necessary to boost the increasing trends and enticing more students in advanced mathematics, advanced science, and CTE-STEM courses.

### Gender disparity was greater in advanced science courses than advanced mathematics courses

While Tyson et al. (2007) showed that female students tended to take low-level mathematics courses, the Texas data revealed that female students took advanced mathematics courses, such as AP Calculus AB and AP Statistics, comparable to or more than male students across 6 years (Fig. 7). Along with the trends that more female students tended to achieve credits for Algebra I and Geometry prior to high school, which are required for high school graduation and prerequisite for advanced mathematics courses, AP Calculus BC was the only advanced mathematics course with lower enrollment rates of female students than male students. This is a positive trend because of the potential to reduce gender disparity in postsecondary STEM majors.

However, the gender bias in course selection was more distinguishable in advance science courses than advanced mathematics courses (Figs. 7 and 8). Figures 7 and 8 on the same scale in percentage show the detectable gender differences in Advanced Biology and Advanced Physics courses, and the differences were steadily increasing across years. While similar proportions of female and male students enrolled in Advanced Chemistry courses across years, more female students (6-year average = 1.8%) enrolled in Advanced Biology courses than male students (6-year average = 1.2%), and vice versa, more male students (6-year average = 1.4%) enrolled in Advanced Physics courses than female students (6-year average = 0.9%) (see Table 9).

These science course preferences in high school seem to continue and may result in the gender disparity even within STEM majors in postsecondary education (Riegle-Crumb, King, Grodsky, & Muller, 2012). For instance, more female students in Advanced Biology may indicate that these students may wish to pursue certain majors in STEM disciplines (e.g., biology, biomedical, and environmental engineering), whereas male students may enroll in Advanced Physics with the intent to major in other fields of study (e.g., physics, aerospace, civil, and mechanical engineering) as shown in bachelor’s degrees conferred by gender and discipline from Digest of Education Statistics 2014 (Snyder, de Brey, & Dillow, 2016, p. 576, Table 318.30).

Based on stereotype threat theory (Steele, 1997), Corra (2007) observed from North Carolina public school data that enrollments of female students in advanced mathematics and physical science courses were comparable to male students and suggested that female students were now less influenced by stereotype threat because of the changing social context. However, our data indicate that Texas high school students may not be free of gendered stereotype threat. Future research on the effects of stereotype and social context on students will warrant understanding of the gender disparity in advanced science courses.

### Collectively, gender gap in CTE-STEM courses increased greater than advanced mathematics and advanced science courses

Even though Figs. 7 and 8 show gender differences in a specific mathematics and science courses, collectively, the gender gaps in advanced mathematics courses and advanced science courses were counterbalanced due to the opposite trends by gender as shown in Fig. 6. However, the gender gaps in CTE-STEM course enrollment rates were not counterbalanced in the collective results and seemed to be increasing across years (6-year average gap = 0.58% for all CTE-STEM courses compared to 0.04% for both advanced mathematics and advanced science courses; see Table 7 for the details). In detail, the gender gaps in Electronics, Principles of Engineering, and Digital Electronics, except Biotechnology and Principles of Technology, were gradually increasing (see Fig. 5).

As our data include all Texas high school students as participants of this study, the trend is similar to the results by Riegle-Crumb and Moore (2013), who explored the gender gap in an upper-level high school engineering course employed by university faculty in six high schools in Texas. They observed a smaller proportion of female students’ enrollment than male students in the course and significant gender gaps in the attitudes toward and perceptions of science and engineering, which were favored by male students.

From the representative national sample, Sadler et al. (2012) observed that (a) STEM career interests were stable by male students but volatile by female students during high school; (b) the STEM interests at the start of high school was a strong predictor of the STEM interest at the end of high school; and (c) there was difficulty in attracting female students to STEM fields during high school. Therefore, shaping STEM interests prior to high school seems to be important to reduce the gender gap at the end of high school. Further research is necessary to identify types of supports for female students to cultivate and sustain interests in STEM from elementary/middle to high school.

### Racial/ethnic differences were constant across years in both advanced mathematics and advanced science courses

The overall enrollments of each race/ethnicity seemed to increase across time in advanced mathematics and advanced science courses, which is a promising phenomenon (see Fig. 12). However, the racial/ethnic gaps were constant across time with the highest enrollments of Asian students (around 15%) and the lowest enrollments of Black students (around 2%) (see Table 12). Similar to Riegle-Crumb’s (2006) finding in the Adolescent Health and Academic Achievement (AHAA) data set, this might be because of the late start of the mathematics course sequence by the underrepresented minority students. The same inference can be made for the advanced science courses as Asian and White students tended to achieve the required mathematics and science credits as early as middle school, so they can enroll/complete the advanced level of the courses during high school.

On the one hand, Riegle-Crumb et al. (2011) showed that eighth grade female students had relatively low mathematics/science career aspirations that differed by racial/ethnic groups. On the other hand, Gilmartin et al. (2006) revealed that the tenth grade students’ interests in physical science and engineering career varied by their race/ethnicity more than gender under the influence of family’s interest in and value of science and their interests were not related to their perceptions of science class experiences. Similarly, focusing on Black students, Corra and Lovaglia (2012) found underrepresentation of Black students, even in high-performing Black students in advanced courses, and tried to explain the phenomena as influence of stereotype threat. Therefore, further investigation is necessary to understand other factors influential to underrepresented populations’ career choice and persistency in STEM in early ages, so they can commit to curriculum tracks for STEM careers during high school under appropriate supporting based on the evidence-based research.

### Racial/ethnic differences were rising in CTE-STEM courses in recent years

While overall increase of the enrollment rates in CTE-STEM courses regardless of gender and race/ethnicity is a promising trend, the recent trend in racial/ethnic gaps is an alarming signal because of the rising gaps among racial/ethnic groups with increasing enrollments of Asian students compared to other racial/ethnic groups (see Fig. 12). This implies that students’ interests in CTE-STEM courses are varied by their racial/ethnic groups due to some reasons, and if there are no efforts made to reduce these racial/ethnic gaps, then the gaps can become wider in the later years. Therefore, this finding recommends actions from educators and education administrators to increase the other racial/ethnic students’ STEM interests.

Tyson et al. (2007) pointed out that “gender disparities in STEM occur because women are less likely to pursue STEM, but racial disparities occur because fewer Black and Hispanic students are prepared for STEM in high school (p. 243)” using Florida data. This study revealed that similar gender and racial/ethnic disparities in STEM course enrollment rates existed in the data from Texas high school students and the results could be interpreted in the same way.

According to the literature, based on the achieved course credits in CTE during high school, students can be classified as coursetakers, investors, and concentrators (Levesque & Hudson, 2003). For example, coursetakers are students who achieved more than 0.0 credits in any CTE areas; investors are students who earned more than 3.0 credits regardless of CTE areas; and concentrators are students who earned more than 3.0 credits in a single CTE area. Using the data of the public high school graduates between 1982 and 1998, Levesque & Hudson, (2003) found that 96.5% of students took at least more than 0.0 credits (coursetakers), followed by 61.5% investors, while 25.0% were concentrators in CTE. With softened criteria for concentrators (who earned 2.0 or more credits in a single CTE area), Levesque et al. (2010) identified 37.6% of concentrators in the class of 2005 high school students and among them, 3.8% earned credits in Computer and Information Science, followed by 2.6% in Engineering Technology, and 2.1% in Construction and Architecture. They also found more White, male, and/or disabled students in concentrators than nonconcentrators. Regarding student performance, Israel et al. (2012) found differentiated student achievement in a standardized science test by the credits in CTE: concentrators with the highest, followed by investors and coursetakers.

As Texas Education Agency (TEA) adopted CTE endorsements in 16 areas from the class of 2018 high school students, House Bill 5 seems to boost the student enrollments in CTE, so we expect to have an increased number of concentrators rather than investors or coursetakers in CTE-STEM, better student performance in standardized science tests, and reduced gaps of enrollments by gender and race/ethnicity subgroups. Note that students can achieve a STEM endorsement by taking more advanced courses in mathematics and/or science. Below is the excerpt from the Subchapter B. Graduation Requirements in Chapter 74. Curriculum Requirements (TEA, 2015) about a STEM endorsement: §74.13. Endorsements – (f)

- A student may earn a STEM endorsement by completing the requirements specified in subsection (e) of this section, including Algebra II, chemistry, and physics and:
  (A) a coherent sequence of courses for four or more credits in career and technical education (CTE) that consists of at least two courses in the same career cluster, including at least one advanced CTE course, which includes any course that is the third or higher course in a sequence. The courses may be selected from Chapter 130 of this title (relating to Texas Essential Knowledge and Skills for Career and Technical Education), Chapter 127 of this title (relating to Texas Essential Knowledge and Skills for Career Development), or CTE innovative courses approved by the commissioner of education. The final course in the sequence must be obtained from one of the CTE career clusters listed in Chapter 130, Subchapter O, of this title (relating to science, technology, engineering, and mathematics); or
  (B) a coherent sequence of four credits in computer science selected from the following: (i) Fundamentals of Computer Science; or (ii) Computer Science I; or (iii) Computer Science II; or (iv) Computer Science III; or (v) Digital Forensics; or (vi) Discrete Mathematics for Computer Science; or (vii) Game Programming and Design; or (viii) Mobile Application Development; or (ix) Robotics Programming and Design; or (x) Independent Studies in Technology Applications; or (xi) AP Computer Science; or (xii) IB Computer Science, Standard Level; or (xiii) IB Computer Science, Higher Level; or
  (C) three credits in mathematics by successfully completing Algebra II and two additional mathematics courses for which Algebra II is a prerequisite by selecting courses from subsection (e)(2) of this section; or
  (D) four credits in science by successfully completing chemistry, physics, and two additional science courses by selecting courses from subsection (e)(5) of this section; or
  (E) in addition to Algebra II, chemistry, and physics, a coherent sequence of three additional credits from no more than two of the categories or disciplines represented by subparagraphs (A), (B), (C), and (D) of this paragraph.

However, considering that a student must earn at least a total of 26 credits with foundational coursework and endorsement in Texas, it is not easy for students who are interested in STEM to commit to both areas of course sequences, CTE-STEM and advanced mathematics and/or science courses. This implies that students need to make a choice in course-taking to focus on one area. Therefore, it is necessary to conduct a longitudinal study on the efficiency and/or effectiveness of the types of STEM endorsements (i.e., mathematics, science, CTE-STEM, and combination of no more than two of the categories) on students’ major choice in STEM and postsecondary performance, and furthermore their career paths in the STEM pipeline.

### Limitations of the study and suggestions for future research

As we utilized the aggregated students’ data from TEA across schools and districts, there are several limitations in data analyses. First, as the data are on the student counts enrolled in the high school courses each year, we could not identify the number of students who already received the high school course credits prior to their high school entrance. Therefore, future work exploring middle school students’ patterns in course-taking or early high school course credit achievement is necessary to understand the effects of early high school coursework in middle school on the future performance in high school and college preparation.

Second, as the data utilized for this study present student enrollment records of all the courses offered in Texas high schools, there is a chance in missing the number of students who enrolled in an online course or a local community college/university during their high school to achieve advanced high school course credits. In addition, while there were some features that stood out in the trends (e.g., a drop of the enrollment rate in 2009 for Algebra II in Fig. 3), we were not able to identify any evidence to support such features in TEA and the literature. Therefore, there is a need for future investigation to explore effects of any historical events on students’ course enrollment.

Third, as we utilized aggregated data, we could not isolate any trends by the effects of school districts, school sizes, and overall school performance. While the literature revealed no significant high school effects on students’ college performance in California (Finkelstein & Fong, 2008), further investigation of the high school effects in high course-taking patterns would be necessary to understand the school dynamics that might influence students’ selection of STEM-related courses.

Fourth, as we were interested in high school students’ interests in STEM-related courses and course-taking patterns, we utilized not student performance data but their enrollment data. Therefore, we could not investigate the direct impact of the STEM-related course-taking on their future performance and connection to the postsecondary STEM pipeline. A longitudinal project, which collects individual students’ data on their performance in the courses and postsecondary status, will warrant understanding of the effects of the STEM-related courses on students’ STEM enrollment at the college level.

Fifth, while there are several STEM-related areas in CTE endorsement tracks, such as (a) Agriculture, Food, and Natural Resources, (b) Architecture and Construction, (c) Information Technology, (d) Manufacturing, and (e) Computer Science, we only explored the enrollment trends in CTE-STEM courses. Therefore, there is a need for future research on the enrollment trends in STEM-related CTE courses and the effects of those courses on students’ postsecondary endorsement and performance in STEM.

Lastly, the student demographics (i.e., gender and race/ethnicity) included in this study provide a limited picture of students’ interests in STEM disciplines at the high school level. The effect of other factors, such as socioeconomic status, parents’ education, and first-generation status, to name as a few examples, are also significant variables worth including for future research.

### Significance of the study

As we have been working on increasing pathways of diverse students pursuing STEM degrees for decades, the findings from this study have several potential merits since little is known about students’ preferences in course-taking in STEM courses at the state level (Long et al., 2012). First, the findings on the trends in students’ STEM course-taking, disaggregated by gender and race/ethnicity, can provide needed insights on what institutional K-12 changes would be effective for impacting the pipeline. Therefore, this study can be used as a stepping stone for research on reasons for the differences in enrollment rates by Texas high school students in CTE courses, along ethnic/racial/gender lines.

Second, even though the scope of the study is a unique case study limited to the high school student population in Texas, there is a potential relevance to extend the findings across the nation, considering the current diversity in Texas. In detail, according to the U.S. Census, 2014 was the critical year of the USA with growing population diversity because it was the first year with the proportion of minority over White. As Texas is one of the largest states with a diverse demographic profile, the current status and issues of Texas education would be relevant to the student population in the future of the nation.

Third, the findings from this study will inform students, parents, teachers, counselors, and administrators in high school as well as policymakers in the state about the efficient high school course preparation that are needed for students to perform successfully in STEM fields in postsecondary. Therefore, the results of this study have broader impact and can inform career counselors and university recruitment efforts to tailor their messaging to students’ behavior of course selection.

Fourth, as this study disclosed the trends of high school students in mathematics, science, and CTE-STEM course enrollments before House Bill 5 in Texas as a baseline, a comparison study will be possible to see the impacts of House Bill 5 implementation on high school students’ course selections in STEM-related areas as the bill is anticipated to have students’ commitment to one specific area through endorsement.

## Footnotes

1 Preliminary findings of the CTE-STEM course enrollments by total, gender and race/ethnicity were reported in a conference paper by Yoon and Strobel (2016).

2 1% of total Texas high school student population ranges from about 13,000 in 2008 and about 14,000 in 2013 according to Table 3.
